# How Little Is Too Much? – How Transient Tumor-Stromal Crosstalk Can Control Tumor Progression

**DOI:** 10.4172/2155-9899.1000462

**Published:** 2016-10-15

**Authors:** Christina H. Stuelten

**Affiliations:** Laboratory of Cellular and Molecular Biology, National Cancer Institute, National Institutes of Health, 37 Convent Drive, Bethesda, MD 20892, USA

**Keywords:** Tumor-stroma interaction, TGF-β, Breast cancer, Dynamical system

## Abstract

Tumorigenesis is driven by genetic and physiological alterations of tumor cells as well as by the host microenvironment. In a co-culture of breast cancer cells and fibroblasts, short term interactions between tumor cells and stromal fibroblasts increase levels of active, fibroblast derived TGF-β in the extracellular medium, which in turn induces an expanded metastatic pattern of MCF10CA1a cells. These findings suggest that the effects of stromal TGF-β on tumor cell phenotype can be modeled as a dynamical system rather than a continuous linear system. In such a model, small changes of certain parameters of a system that is at a critical point can cause sudden changes of the system, explaining why experimentally and clinically observed small changes in the tumor environment can cause dramatic changes in cell phenotype or disease outcome.

## Commentary

Tumorigenesis is driven by genetic and physiological alterations of tumor cells as well as by the host microenvironment [1]. The stromal microenvironment was first implicated as a critical player during tumor development by Virchow when he postulated in 1863 that chronic inflammation could cause cancer [2]. Indeed, the local stroma which is composed of acellular matrix, interstitial fluid and stromal cells such as fibroblasts, endothelial cells and immune cells, can support or suppress tumor growth in many ways [1,3]. First identified as cells that merely provide extracellular matrix as scaffolding for tissues and tumors, fibroblasts are now recognized to be involved in the reciprocal tumor-stromal crosstalk and tumor development via secretion of cytokines, growth factors and enzymes, or modification of the extracellular matrix [3,4]. Unexpectedly, a recent study showed that even transient interactions between tumor cells and fibroblasts can significantly impact tumor progression through a mechanism that is dependent on TGF-β [5]. The underlying mechanisms and conceptual implications of this work are discussed below.

TGF-β is a pleiotropic cytokine that can act as a tumor suppressor or a metastasis promotor [6]. TGF-β is synthesized and secreted in a biologically latent form into the local microenvironment where it can be activated by integrin binding, by matrix metalloproteinases, or by pH changes [7]. Active TGF-β then binds to specific TGF-β receptors and subsequently activates Smad and MAPK signaling cascades [8]. Effects of TGF-β are cell type- and context-dependent. In tumors, TGF-β is an important mediator of tumor stromal crosstalk and has been implicated in attracting immune and endothelial cells to the tumor, to activate fibroblasts and to alter tumor cell biology [9].

Co-culture systems have illuminated interesting aspects of this cross-talk. In a model of breast cancer, co-cultures of normal fibroblasts and breast cancer cells (MCF10CA1a) secrete small amounts of active TGF-β (0.1–0.2 ng/ml) into the extracellular medium, an effect that is not seen when either cell type is cultured separately [5]. Use of TGF-β1 knockout fibroblasts showed that the fibroblast-derived TGF-β in the conditioned medium causes naïve tumor cells to scatter and to change E-Cadherin expression patterns *in vitro*. More importantly, short-term exposure of naïve MCF10CA1a cells to co-culture medium (CoCM) *in vitro* leads to sustained TGF-β signaling and an expanded metastatic pattern in an orthotopic xenograft mouse model *in vivo*. This effect is TGF-β dependent, implying that the TGF-β that is made or activated by fibroblasts when they are temporarily exposed to tumor cells, can durably increase the metastatic potential of tumor cells.

The change in phenotype upon stimulation of MCF10CA1a cells with CoCM containing active TGF-β is reminiscent of the effect of morphogens which act in specific concentration ranges [10]. Indeed, TGF-β has been described as a tubulogen in a 3D model of mammary tubulogenesis, where TGF-β concentrations of 20 pg/ml to 100 pg/ml induce mammary tubulogenesis *in vitro* [11]. However, tubulogenesis is increasingly disturbed if TGF-β concentrations are greater than 200 pg/ml [11]. At 200–500 pg/ml TGF-β pointed outgrowths that lack a visible lumen are observed, and concentrations greater than 500 pg/ml lead to disorganized, lumen-less cell aggregates from which streaks of cells grow into the surrounding matrix. Thus, increasing extracellular levels of active TGF-β can induce an abrupt transition or switch to disordered growth if a critical TGF-β concentration is exceeded.

The experiment described above allows to predict that more animals injected with CoCM treated MCF10CA1a cells will develop extrapulmonary tumors than animals injected with control cells. However, it is not possible to predict which specific animal will develop such a tumor, and when the tumor will develop. This uncertainty of outcome, as well as the abrupt change of observed phenotype in some animals after stimulation of tumor cells with CoCM, implies that the observed effect may best be described as a dynamical system. In dynamical systems a “bifurcation” occurs if small changes in a parameter cause a sudden qualitative change of the system. As the parameters included in the system change, different, possibly metastable, states are possible. Applied to the co-culture system discussed, this implies that as a tumor cell is exposed to changing concentrations of TGF-β it will retain its initial state until a critical concentration of TGF-β is reached. At this critical concentration, the system will go through a bifurcation, and even small changes of TGF-β levels - the bifurcation parameter - can cause the system to transition to a second state - an expanded metastatic pattern.

Another biological phenomenon that has been modeled as a dynamical system is the epithelial mesenchymal transition (EMT) [12,13]. In this model of epithelial-mesenchymal fate determination, a miR-34 / SNAIL and mir-200/ZEB circuit is used to model three metastable states - epithelial, mesenchymal, and epithelial / mesenchymal - and transitions between these states. The viral hit and run oncogenesis model [14] is another example of a dynamical system. In this model a viral infection transiently transforms a cell (hit) before it is eliminated from the genome (run) leaving a permanently altered and malignant cell behind. This concept is particularly intriguing for viruses that can abruptly alter the expression or activity of enzymes (e.g. DNA methyltransferases as is described for HBV, HCV and HPV) and consequently cause lasting epigenetic changes [14]. Here, the DNA methyltransferase activity would be a critical parameter, and as critical levels are reached the subsequent epigenetic changes may result in a new phenotype even in the absence of viral DNA. Other examples for sudden qualitative changes of biological phenotypes that could be modeled by a dynamical system are (i) induction of tumor growth in non-tumor bearing areas in Rous sarcoma virus infected chicken by wounding or TGF-β [15], (ii) tumor stem cell fate decisions, or (iii) clinically observed but unexplained spontaneous tumor regression.

How little of a disturbance is enough to cause transition of a tumor to the next stage? Applying dynamical systems modeling can conceptually answer this question: If a parameter x, for example TGF-β concentration, that drives tumor progression is at critical level - a bifurcation point - even the smallest change can cause a sudden shift of the system or tumor to a new state or tumor stage. In contrast, a similar sized disturbance may not have any perceived effect on the system if it occurs at a different point. This may well be the situation for normal cells that typically do not respond to acute physiological stimuli like wounding or inflammation by abruptly transitioning to malignant growth. Thus, if a tumor is at a critical point, even the smallest change is too much to maintain the current state and will alter the disease course.

In conclusion, short term interactions of tumor cells and stromal fibroblasts increase levels of active, fibroblast derived TGF-β in the extracellular medium, which in turn induces an expanded metastatic pattern of MCF10CA1a cells. This abrupt change in metastatic phenotype after short-time exposure to increased TGF-β levels is evidence that the cell fate of tumor cells is determined by a multi-parameter dynamical system rather than a continuous linear system. Therefore, apparently small and temporary changes of specific parameters can cause abrupt and significant changes in the course of a disease such as cancer.
